# Microbial musings – April 2020

**DOI:** 10.1099/mic.0.000925

**Published:** 2020-04-30

**Authors:** Gavin H. Thomas

**Affiliations:** ^1^​ Department of Biology, University of York, York, YO10 5YW, UK

As we continue lockdown in the UK and across Europe and most of the globe, the working day of the academic microbiologist is adapting to laboratory closures and a desire to try and help out at a local or national level where possible. Many members of the Microbiology Society, including *Microbial Genomics* editor Alan McNally (@alanmcn1) and microbiologists from Birmingham and the Midlands, have been involved in the Lighthouse Lab, set up in Milton Keynes, where they are offering their time and expertise to increase testing. Two other large diagnostic labs are opening in Alderley Park and Glasgow shortly and I am sure many Microbiology Society members are helping out with this. Other microbiologists are helping to develop vaccines and understand the structure and function of viral proteins. In York, where we are too far from any large new testing centre, our lab has been turned into an assembly line for personal protection equipment (PPE), piecing together community 3D printed visors for distribution to regional healthcare workers in hospitals and the community. These efforts are being mirrored in other centres across the UK and more widely as we all try and find ways to help. It is brilliant seeing our community stepping up to the challenges in the UK and around the world.

We start this month with a fascinating review by Dave Clarke from Cork, Ireland (@cleverflick) about the genus *Photorhabdus* [1]. We introduced a number of classical symbionts last month [2], but *Photorhabdus* is more complicated than this in terms of its particular mutualism. These Gram-negative proteobacteria have attracted research interest because of their interesting lifestyle and because they are a potential source of biocontrol agents. In young nematodes they are commensals in the animal’s gut, but the nematodes they live in association with have a propensity to infect the larva of insects such as beetles, flies and butterflies. This is enabled by their bacterial ‘partners in crime’, who are released into the insect haemolymph, where they grow and release toxins that kill the insect. The nematodes then digest the dead larva and reproduce and the bacteria recolonize the young nematodes when they leave the cadaver to seek out new prey. The review covers research that is revealing the molecular basis of the interactions between *Photorhabdus* and the insect, where *Galleria mellonella* larvae are used as a model system, as well as the interactions with the nematode host. Much of this research has focused on how the bacterium senses the right time to secrete a suite of toxins to kill the insect. One unusual aspect of this armoury is the use of phage-like particles by the bacterium to deliver payloads into the insect cells, a story elucidated by Nick Waterfield (@Nick_Waterfield) and colleagues in Warwick with long-time *Photorhabdus* researcher Richard ffrench-Constant. The genus has also been in the news recently as a member of a new class of antibiotics called darobactin, which is active against Gram-negative pathogens, and was discovered late last year from a screen of *Photorhabdus* isolates [3].

Sticking with antibiotics, a nice paper from Martha Ramiraz-Diaz and colleagues at the Universidad Michoacana de San Nicolás de Hidalgo, Morelia, Mexico, examines the biochemical workings of a relatively new protein involved in resistance to fluoroquinolones [4]. Whilst mechanisms for fluoroquinolone resistance are well known through alteration of target sites in DNA gyrase and topoisomerase and also through reduced permeability and efflux, this study is on a protein, CrpP, from a clinical strain of *Pseudomonas aeruginosa *, that directly modifies the antibiotic by phosphorylation. Antibiotic modification is well known for other classes of antibiotics, such as aminoglycosides, but less well known for fluoroquinolones. In this study the authors take a structure/function approach by mutating a number of residues in CrpP that they predict will be important for the catalytic function of the enzyme, which they test both *in vivo* and *in vitro*. While no structures are available for these small proteins, the work highlights a number of conserved residues that are essential for resistance and ciprofloxacin modification and are probably important for ATP binding and phosphotransfer to the antibiotic.

We switch now to Gram-positive bacteria, with a series of papers linked in some way through the function of small peptides/proteins in bacterial physiology. There are some classic stories from early days of molecular microbiology that are underpinned by the function of small secreted peptides, like the signals required for sporulation in *Bacillus subtilis* or the lovely work of Gary Dunny’s group in Minnesota, USA, on the ‘sex pheromone’ peptide used to induce conjugation in *Enterococcus faecalis* [5]. For these small peptides to function in signalling they need to be synthesized, usually from cleavage of longer polypeptides, secreted, retaken up and then detected. Uptake of these peptides requires use of either general or specific peptide uptake systems, usually ABC transporters. For example, one of the early sporulation-defective mutants of *Bacillus subtilis *, the *spo0K* strain, was found to be inactivated in its oligopepide uptake system [6] and only later was its role in the import of the quorum-sensing Phr peptides discovered [7].

In this issue a quorum sensing-related peptide signalling system from *Bacillus thuringiensis* and *Bacillus cereus* is characterized further by the group of Eugenie Huillet and Didier Lereclus from the University of Paris-Saclay in France [8]. In this system the heptapeptide PapR is known to be the main signal for the important virulence-related transcription factor (TF) PlcR, and once taken up by the oligopeptide ABC transporter binds directly to a domain in PlcR, allowing it to interact with its target promoters. In this paper the authors examine a second related TF called PlcRa, which is also present in *B. thuringiensis* and they discover can also be activated by the PapR peptide, despite this not being its ‘cognate’ sensory peptide. The authors use a range of genetic and structural methods to examine this signalling crosstalk to show that a single signalling peptide can be used to functionally connect multiple regulons in *B. thuringiensis* and *B. cereus *.

A second paper, which was strictly published in the March issue, from Robert Burne’s group at the University of Florida, USA, is on the function of two small peptides in *Streptococcus mutans* [9] that are encoded at the back end of an operon containing ABC efflux protein genes. In contrast to the stories of small peptides being secreted and functioning in sensory roles, there are also small peptides that perform a wide range of other functions inside the cell. This has interested me for a long time, in fact ever since I was involved in the reannotation of the *Escherichia coli* K12 genome in 2006 [10] when chats with Ken Rudd during our annotation meetings at Wood’s Hole, USA, led me to learn about Gigi Storz’s (@storzlab) amazing work finding small peptides encoded on bacterial genomes and figuring out their function [11]. Notable examples that are now well understood are the SgrT protein involved in carbohydrate metabolic regulation characterized by Cari Vanderpool’s lab [12] (@MicroPhysIL) and a very interesting small transmembrane peptide AcrZ that interacts with the important drug efflux pump AcrAB [13]. In the current paper the authors introduce the novel peptides that they discovered in 2014 with a role in regulating competence development [14] and continue to study their function within the *rcrRPQ* operon in which they are located. They find additional functions for this operon in thermotolerance, which requires both the efflux pumps and the small peptides. They further show that another small peptide, XrpA, is also involved in this process, as its deletion can restore thermotolerance to *S. mutants* mutants. Clearly this is a complicated system, but it has likely functions for three small peptides, perhaps in allosteric regulation of other proteins.

The third paper related to the peptide theme is about the activation of another two-component regulator in *Streptococcus pneumoniae *, from the group of Reinhold Bruckner’s in Kaiserslautern, Germany [15]. This protein CiaR controls a series of genes that together are involved in damping down the effect of the competence-stimulating peptide (CSP) through catalyzing its rapid degradation or reducing its expression. In this paper the authors make a very interesting discovery about an additional route by which CiaR is activated. Normally CiaR is phosphorylated by its cognate histidine kinase protein, CiaH, but when this is deleted it can still be activated by another phosphor donor, namely acetyl phosphate. It has been known for many years that acetyl-phosphate can do this with many different response regulators [16], but here the authors demonstrate a direct protein–protein interaction between acetate kinase (AckA) and CiaR, using the *E. coli* two-hybrid system. This is highly intriguing as AckA is the enzyme that can both synthesize and degrade acetyl-phosphate, depending on the particular physiological conditions, and hence is key in controlling intracellular acetyl-phosphate levels. The authors test the idea that associating with AckA could cause dephosphorylation of CiaR, but this is not seen experimentally and they come up some other hypotheses as to what the purpose of this interaction might be. If this interaction is conserved beyond *S. pneumoniae* and also to other response regulators, then it could add another level of complexity to the role of acetyl-phosphate in cellular physiology.

Finally, a flag for our Editor’s Choice paper for the April issue, selected by senior editor Hana Sychrova, which is a paper looking at a bacterial infection of *G. mellonella* larvae [17]. We have already seen these model insect larvae being used to learn about *Photorhabdus *, but in this paper the authors identify another inter-domain interaction between the yeast *Candida albicans* and the bacterium *Staphylococcus aureus *. Read Hana’s commentary on this in April’s Microbepost over at the main Microbiology Society site. Stay safe and see you next month for more papers from *Microbiology*.
